# Supplementary material for: Enantioselectivity of Pentedrone and Methylone on Metabolic Profiling in 2D and 3D Human Hepatocyte-like Cells
Source: Pharmaceuticals (Basel). 2022 Mar 17;15(3):368. doi: 10.3390/ph15030368 (PMC8953427; doi:10.3390/ph15030368)
Supplement: Supplementary file 1 [file pharmaceuticals-15-00368-s001.zip › pharmaceuticals-1600972-supplementary.pdf]

Supplementary Materials

Enantioselectivity of Pentedrone and Methylone on Metabolic Profiling in 2D and 3D Human Hepatocyte-like Cells

Bárbara Silva [1,2,3,*], Joana Saraiva Rodrigues [4], Ana Sofia Almeida [3], Ana Rita Lima [1,2], Carla Fernandes [3,5], Paula Guedes de Pinho [1,2], Joana Paiva Miranda [4,†] and Fernando Remião [1,2,*,†]

1 Associate Laboratory i4HB—Institute for Health and Bioeconomy, Faculdade de Farmácia, Universidade do Porto, Rua Jorge Viterbo Ferreira, 228, 4050-313 Porto, Portugal; ritacmlima@hotmail.com (A.R.L.); pguedes@ff.up.pt (P.G.d.P.)
2 UCIBIO-REQUIMTE, Laboratório de Toxicologia, Departamento de Ciências Biológicas, Faculdade de Farmácia, Universidade do Porto, Rua Jorge Viterbo Ferreira, 228, 4050-313 Porto, Portugal
3 Laboratório de Química Orgânica e Farmacêutica, Departamento de Ciências Químicas, Faculdade de Farmácia, Universidade do Porto, Rua de Jorge Viterbo Ferreira, 228, 4050-313 Porto, Portugal; up201605043@edu.ff.up.pt (A.S.A.); cfernandes@ff.up.pt (C.F.)
4 Research Institute for Medicines (imed), Faculdade de Farmácia, Universidade de Lisboa, Avenida Professor Gama Pinto, 1649-003 Lisboa, Portugal; joana.s.rodrigues@campus.ul.pt (J.S.R.); jmiranda@ff.ulisboa.pt (J.P.M.)
5 Centro Interdisciplinar de Investigação Marinha e Ambiental (CIIMAR), Universidade do Porto,
Avenida General Norton de Matos, 4450-208 Matosinhos, Portugal
* Correspondence: barbarapolerisilva@gmail.com (B.S.); remiao@ff.up.pt (F.R.)
† These authors contributed equally to this work.

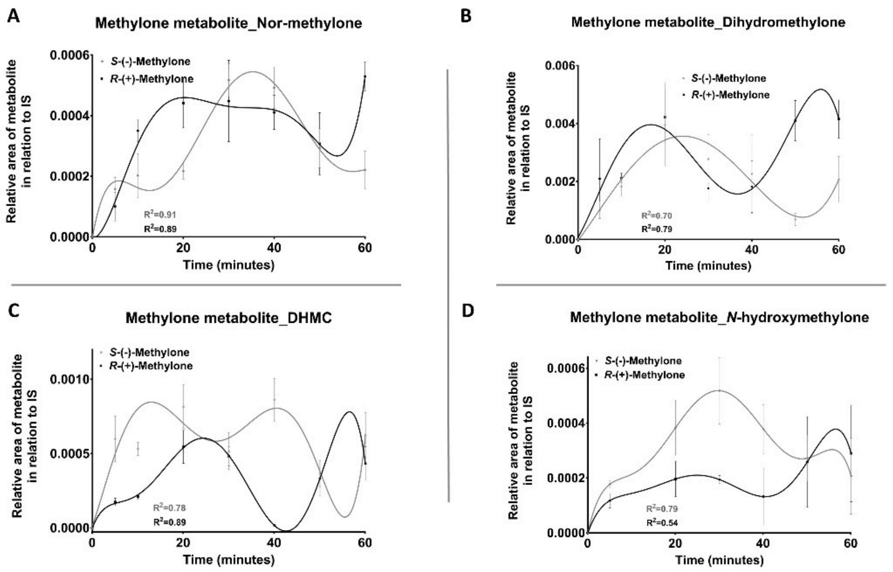

Figure S1. Methylone metabolites (nor-methylone, DHMC, *N*-hydroxymethylone and dihydromethylone) produced over the time after incubation with microsomes (1 mg/mL). Data is presented as average ± SD (n=2). Polynomial regression was used to fit the curves (Nor-methylone, DHMC and *N*-hydroxymethylone: Centered sixth order; Dihydromethylone: Centered fifth order).

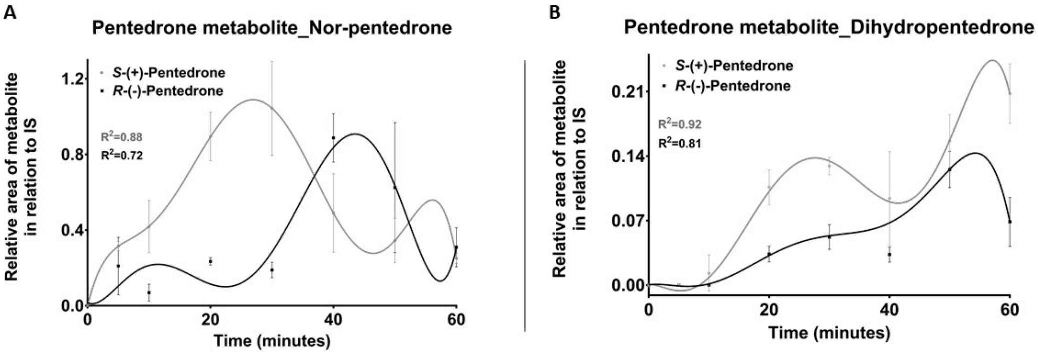

Figure S2. Pentedrone metabolites (nor-pentedrone and dihydropentedrone) produced over the time after incubation with microsomes (1 mg/mL). Data is presented as average ± SD (n=2). Centered sixth order polynomial regression were used to fit the curves.

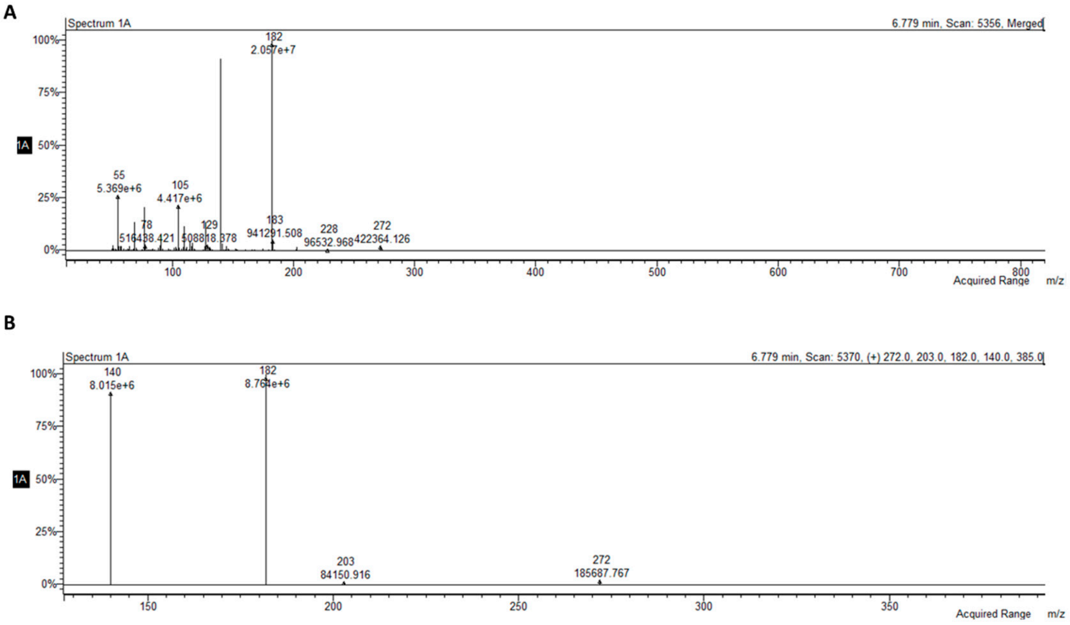

Figure S3. GC-MS spectrum of dihydropentedrone in (**A**) Full Scan and (**B**) SIM mode.

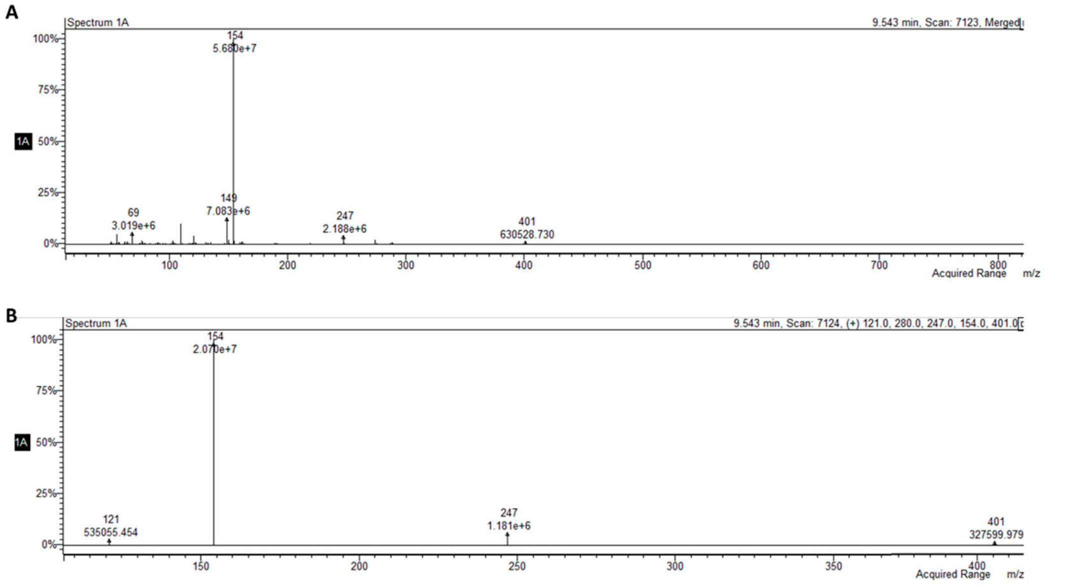

Figure S4. GC-MS spectrum of dihydromethylone in (**A**) Full Scan and (**B**) SIM mode.

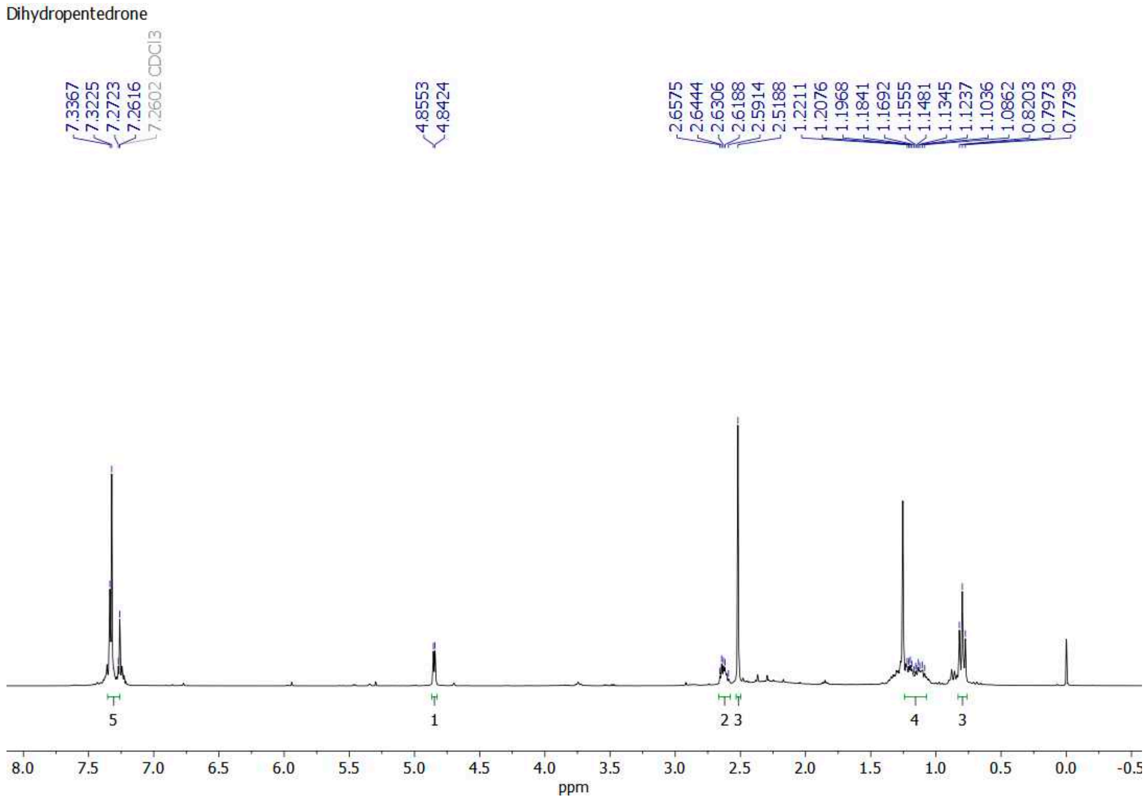

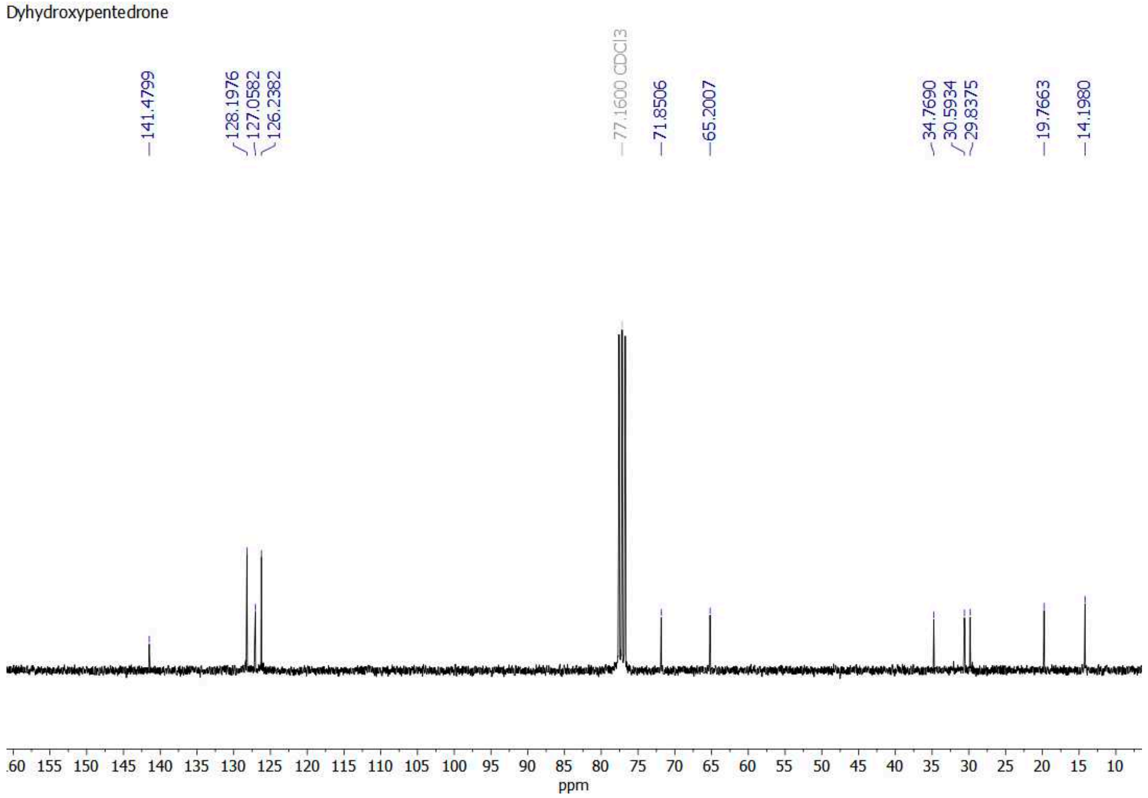

Figure S5. ^{1}H NMR (300.13 MHz, $CDCl_3$) and ^{13}C NMR (75.48 MHz, DMSO-d_6) for dihydromethylone.

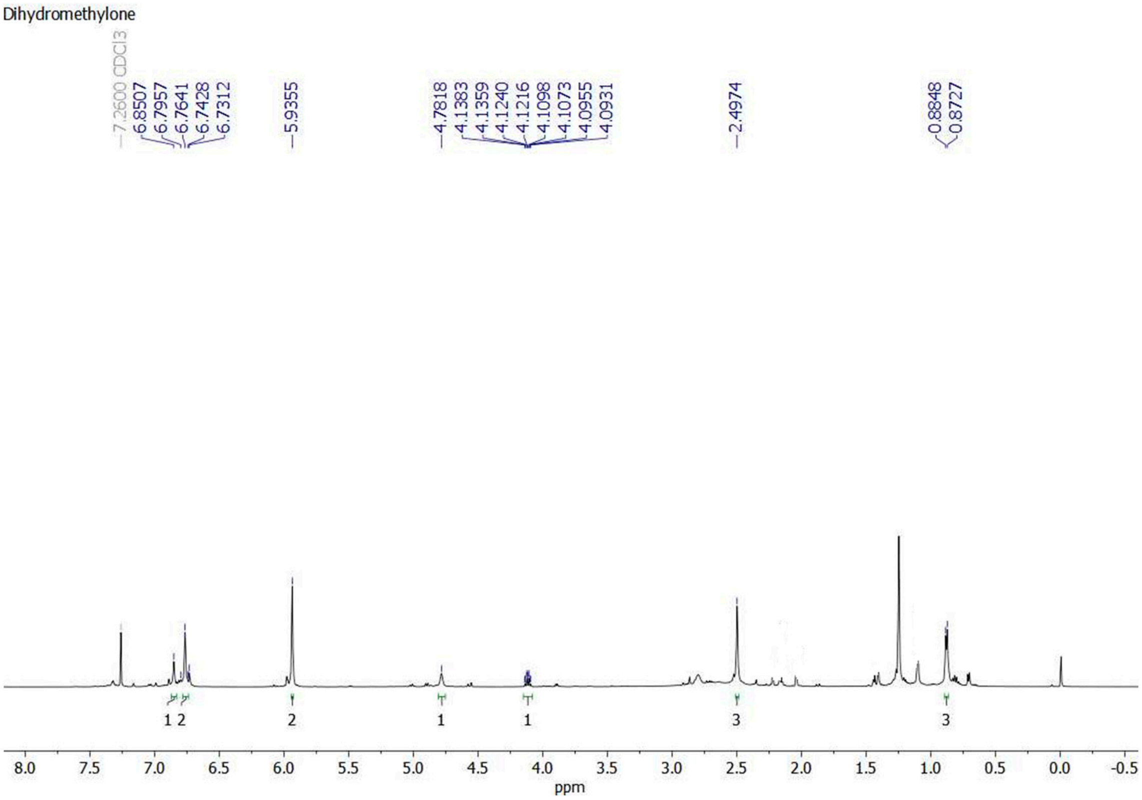

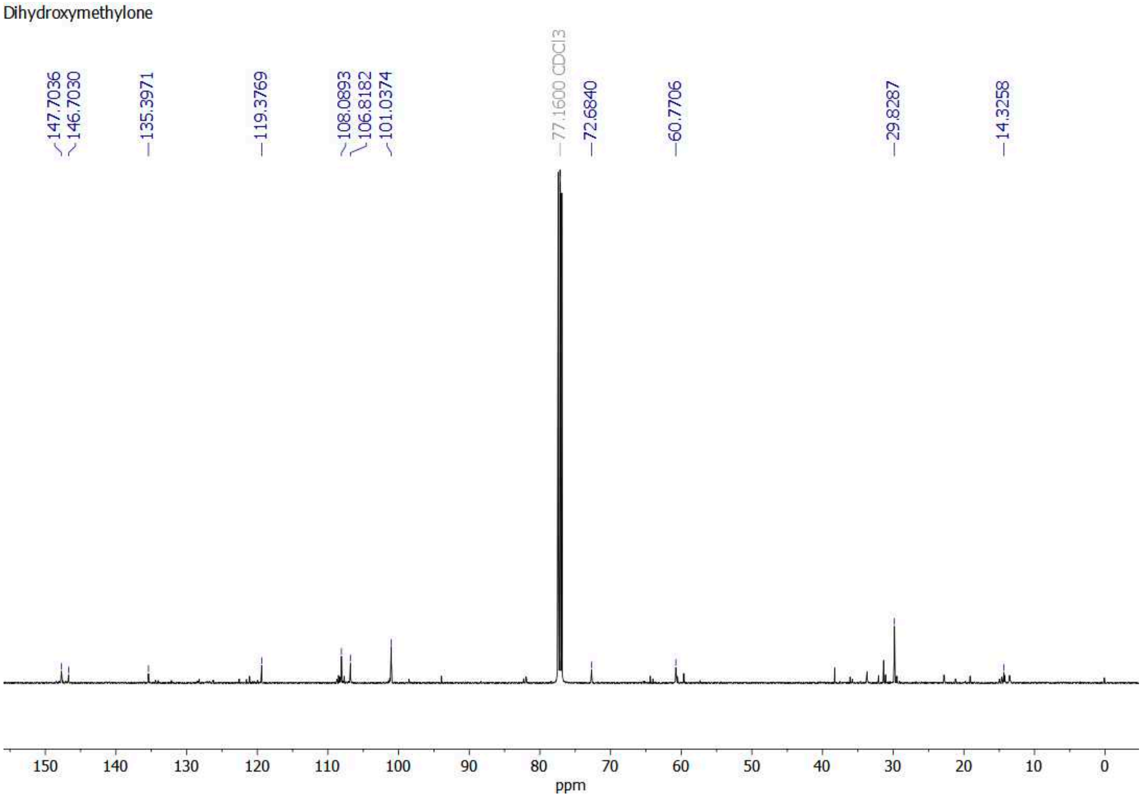

Figure S6. ^{1}H NMR (300.13 MHz, $CDCl_3$) and ^{13}C NMR (75.48 MHz, DMSO-d_6) for dihydropentedrone.
